# Does serum CA125 have clinical value for follow-up monitoring of postoperative patients with epithelial ovarian cancer? Results of a 12-year study

**DOI:** 10.1186/s13048-017-0310-y

**Published:** 2017-03-11

**Authors:** Na Guo, Zhilan Peng

**Affiliations:** Department of Obstetrics and Gynecology, Key laboratory of Birth Detects and Related Diseases of Women and Children(Sichuan University), Ministry of Education, West China Second University Hospital, Sichuan University, Chengdu, 610041 China

**Keywords:** Epithelial ovarian cancer, Serum CA125, Follow-up, Recurrence

## Abstract

**Background:**

The detection of CA125 has been used in the follow up of ovarian cancer. At present, some scholars believe that serum CA125 has no clinical value for the follow-up monitoring the recurrence for postoperative patients with epithelial ovarian cancer, but in our clinical follow-up found that when the serum CA125 value is <35 U/ml, postoperative patients of epithelial ovarian carcinoma had already showed recurrent lesions in some ecological and imaging examinations or in laparotomy exploration and biopsy, and we given the patients timely treatment, the prognosis were improved.

**Methods:**

Retrospective analysis the values of serum CA125 of 342 postoperative patients of epithelial ovarian carcinoma, consisting of 296 non-recurrent and 46 recurrent cases, as well as 3175 cases of menopausal women and 603 cases of postoperative patients of gynecological malignant tumor for the follow-up from January 2005 to December 2016.

**Results:**

The median value of CA125 for non-recurrent patients of epithelial ovarian carcinoma is 8.9 U/ml, the median value of CA125 for non-recurrent patients of epithelial ovarian carcinoma is 29.7 U/ml; for menopausal women, 8.1 U/ml; and for postoperative patients of gynecological malignant tumor, 7.2 U/ml, whereas the mean ± standard deviation is 9.0 ± 1.9 U/ml, 31.3 ± 16.2U/ml, 8.0 ± 1.1 U/ml, and 6.8 ± 2.1 U/ml, respectively.

**Conclusions:**

If the value of the CA125 for postoperative patients of epithelial ovarian carcinoma between 10 and 35 U/ml indicates a relative risk of recurrence. When the value of CA125 is higher than 10 U/ml and continuously increased, need to be vigilant and should be combined with imaging examination (PET-CT). This result may improve the prognosis for recurrent patients because of the early detection of recurrent lesions and early retreatment.

## Background

CA125 is a glycoprotein antigen recognized using OVCA433 as the antigen for preparing the murine monoclonal antibody OC125 in epithelial ovarian carcinoma. It was first reported by the American scientist Bast in 1981 [1]. The detection of CA_125_ has become an effective way to diagnose ovarian epithelial carcinoma and to monitor its condition changes over the years. In 2003, the Gynecologic Oncology Branch of the Chinese Medical Association developed the following standard indicators for the diagnosis and treatment of recurrent ovarian malignant tumor: (1) increased tumor markers, (2) appearance of ascites and pleural effusion, (3) mass revealed by physical examination, (4) tumor revealed by imaging examination, and (5) unknown cause of intestinal obstruction. The presence of one indicator suggests recurrence, whereas the presence of two or more indicates tumor recurrence [2].

Observation of the dynamic change in CA125 to monitor the recurrence of epithelial ovarian carcinoma is widely used in clinics, At present, some scholars believe that serum CA125 has no clinical value for the follow-up monitoring the recurrence for postoperative patients with epithelial ovarian cancer [3], they established the benefits of early treatment on the basis of increased CA125 concentrations compared with delayed treatment on the basis of clinical recurrence, the result showed no evidence of a survival benefit with early treatment of relapse on the basis of a raised CA125 concentration alone. In their treatment, the patients were only treated by chemotherapy, but not surgery. It is known that ovarian cancer is mainly treated by surgery, our clinical follow-up found that when the serum CA125 value is <35 U/ml, some postoperative patients of epithelial ovarian carcinoma who underwent cytoreductive or radical surgery and chemotherapy show recurrent lesions in gynecological and imaging examinations or in laparotomy exploration and biopsy, early detected the lesions, the patients had the opportunity for surgery, the prognosis were improvement and the survival time were prolonged.

The present study aims to observe the dynamic monitoring of serum CA125 values in postoperative patients of epithelial ovarian carcinoma with timely imaging examination, early detection of small recurrent lesions, early treatment, improvement of treatment effect, and prolonged survival time.

## Methods

The subjects of this investigation are 342 patients treated from January 2005 to December 2016 who have been diagnosed with epithelial ovarian carcinoma by pathology examination and have been detected with serum CA125 during and after chemotherapy. The patients’age ranges from 19 to 83 years old, and the median age is 50 years old. The follow-up time is 12–144 months, and the median time is 76 months. Of the 342 subjects, 192 had serious cystadenocarcinoma;26,mucinous cystadenocarcinoma; 32, endometrial adenocarcinoma; 23, clear cell carcinoma; 66, mixed type cases; and 3, undifferentiated carcinoma. The criteria and guideline for surgical pathology staging were provided by the International Federation of Gynecology and Obstetrics (FIGO) (Table 1). Seven cases involving young patients opted to retain their reproductive functions, whereas the rest underwent satisfactory cytoreductive surgery (residual tumor lesions ≤1 cm) and received platinum-based chemotherapy after. Each patient regularly underwent CA125 test, gynecological examination, abdominal ultrasound, chest radiograph, or CT/MRI/PET examinations before and after chemotherapy.

**Table 1:** Pathological type distribution of epithelial ovarian carcinoma patients

Category	Pathology staging				
	I	II	III	IV	Total
Serous cystadenocarcinoma	15(7.8)	23(12.0)	145(75.5)	9(4.7)	192(56.1)
Mucinous cystadenocarcinoma	9(34.6)	3(11.5)	14(53.9)	0(0)	26(7.6)
Endometrial adenocarcinoma	11(34.4)	5(15.6)	16(50.0)	0(0)	32(9.4)
Clear cell carcinoma	19(82.6)	0(0)	4(17.4)	0(0)	23(6.7)
Mixed type and undifferentiated carcinoma	26(37.7)	24(34.8)	19(27.5)	0(0)	69(20.2)
Total	80(23.4)	55(16.1)	198(57.9)	9(2.6)	342(100.0)

Three thousand one hundred seventy-five cases of menopausal women without complications were selected, whereas 603 postoperative patients of gynecological malignant tumor, except epithelial ovarian tumor, were chosen as the control group. The menopausal women’s age ranges from 50 to 88 years old, and the median age is 59 year old. The postoperative patients’ age ranges from 26 to 73 years old, and the median age is 50. The follow-up time is 12–144 months, and the median time is 76 months.

The levels of CA125 for all patients before operation were all >35 U/ml and received regulated the platinum-based combination chemotherapy after operation, regular follow-up, and monthly monitoring of CA125, every 3 months accepted clinical and imaging examinations. Blood samples measuring 2 ml were used as separation serum. Automatic chemiluminescent immunoassay analyzer Siemens Centaur XP auxiliary reagent was used to detect the concentration of CA125, observing the dynamic changes in CA125 in gynecological and imaging examination. SPSS 13.0 software was used for statistical analysis. Count data were analyzed by *χ*2 test, with *P* <0.05 considered statistically significant.

## Results

1. Value distribution of serum CA125 in postmenopausal women

The range of serum CA125 in 3175 cases of menopausal women is 2–29.1 U/ml, the median is 8.1 U/ml, and the mean ± standard deviation is 8.0 ± 1.1 U/ml (Table 2). The CA125 ≤ 10 U/ml in the total proportion of postmenopausal women is 3134/3175 (98.7%).

**Table 2:** Level of serum CA_125_ in postmenopausal women and the patients of each group

CA_125_ level(U/ml)	Menopausal women(*N* = 3175)	Epithelial ovarian carcinoma patients		Non-recurrent patients of gynecological malignant tumor
		Non-recurrent (*N* = 296)	Recurrent (*N* = 46)	(*N* = 603)
Rang	2 ~ 29.1	2.0 ~ 34.9	10.2 ~ 82.8	4.1 ~ 20.8
Median	8.1	8.9	29.7	7.2
X ± S	8.0 ± 1.1	9.0 ± 1.9	31.3 ± 16.2	6.8 ± 2.1

2. Value distribution of serum CA125 in non-recurrent postoperative patients of gynecological malignant tumor

There were 603 cases of non-recurrent postoperative patients of gynecological malignant tumor, except epithelial ovarian tumor patients who were ovariectomized. The follow-up time is 12–144 months, and the median time is 76 months. From this group, 33 had fallopian tube cancer; 167, endometrial cancer; and 403, cervical cancer (Table 3). The range of CA125 is 4.1–20.8 U/ml, the median is 7.2 U/ml, and the mean ± standard deviation is 6.8 ± 2.1 U/ml(Table 2).

**Table 3:** Value distribution of serum CA_125_ in ovariectomized, non-recurrent, postoperative patients of gynecological malignant tumor

Type	Pathology staging				Total	CA_125_ level(U/ml)		
	I	II	III	IV		Range	Median	X ± S
Fallopian tube cancer	3	7	17	6	33(5.5)	4.1 ~ 12.0	7.4	8.1 ± 2.4
Endometrial cancer	55	88	21	3	167(27.7)	3.1 ~ 15.1	7.0	7.3 ± 2.8
Cervical cancer	152	200	34	17	403(66.8)	3.5 ~ 20.8	6.6	6.7 ± 2.2
Total	210	295	72	26	603(100)	4.1 ~ 20.8	7.2	6.8 ± 2.1

3. Value distribution of serum CA125 in non-recurrent postoperative patients of epithelial ovarian carcinoma

There were 296 cases of non-recurrent patients in the total proportion of epithelial ovarian carcinoma (86.5%). The follow-up time is 12–144 months, and the median time is 76 months. The dynamics of changes in CA125 levels is 2–34.9 U/ml, the median is 8.9 U/ml, and the mean ± standard deviation is 9.0 ± 1.9 U/ml(Table 2). Statistical analysis of the CA125 values of non-recurrent postoperative epithelial ovarian carcinoma patients and postmenopausal women and the non-recurrence of gynecological malignant tumor patients showed no significant difference (*P* >0.05). The postoperative CA125 values were divided into four groups:≤10 U/ml, 10–20 U/ml, 20–35 U/ml, and ≥35 U/ml. There were 275 cases of epithelial ovarian carcinoma non-recurrent patients whose CA125 ≤ 10 U/ml (275/296, 92.9%) (Table 4).

**Table 4:** Value distribution of serum CA_125_ in postoperative patients of epithelial ovarian carcinoma

CA_125_ value(U/ml)	Non-recurrent				Recurrent;			
	N (%)	Range	Median	X ± S	N (%)	Range	Median	X ± S
10≥	275(92.9)	2 ~ 10	6.9	6.2 ± 1.8	(0)0	_	_	_
20 ~ 10	15(5.1)	10.3 ~ 16.4	14.1	12.9 ± 2.1	16(34.8)	10.2 ~ 18.9	15.3	14.2 ± 2.5
35 ~ 20	6(2.0)	25 ~ 34.9	27.1	26.7 ± 3.1	15(32.6)	21.1 ~ 34.8	24.4	24.2 ± 3.4
35<	(0)0	_	_	_	15(32.6)	35.1 ~ 82.8	46.8	49.6 ± 11.9

4. Value distribution of serum CA125 in recurrent postoperative patients of epithelial ovarian carcinoma

**Fig. 1:**
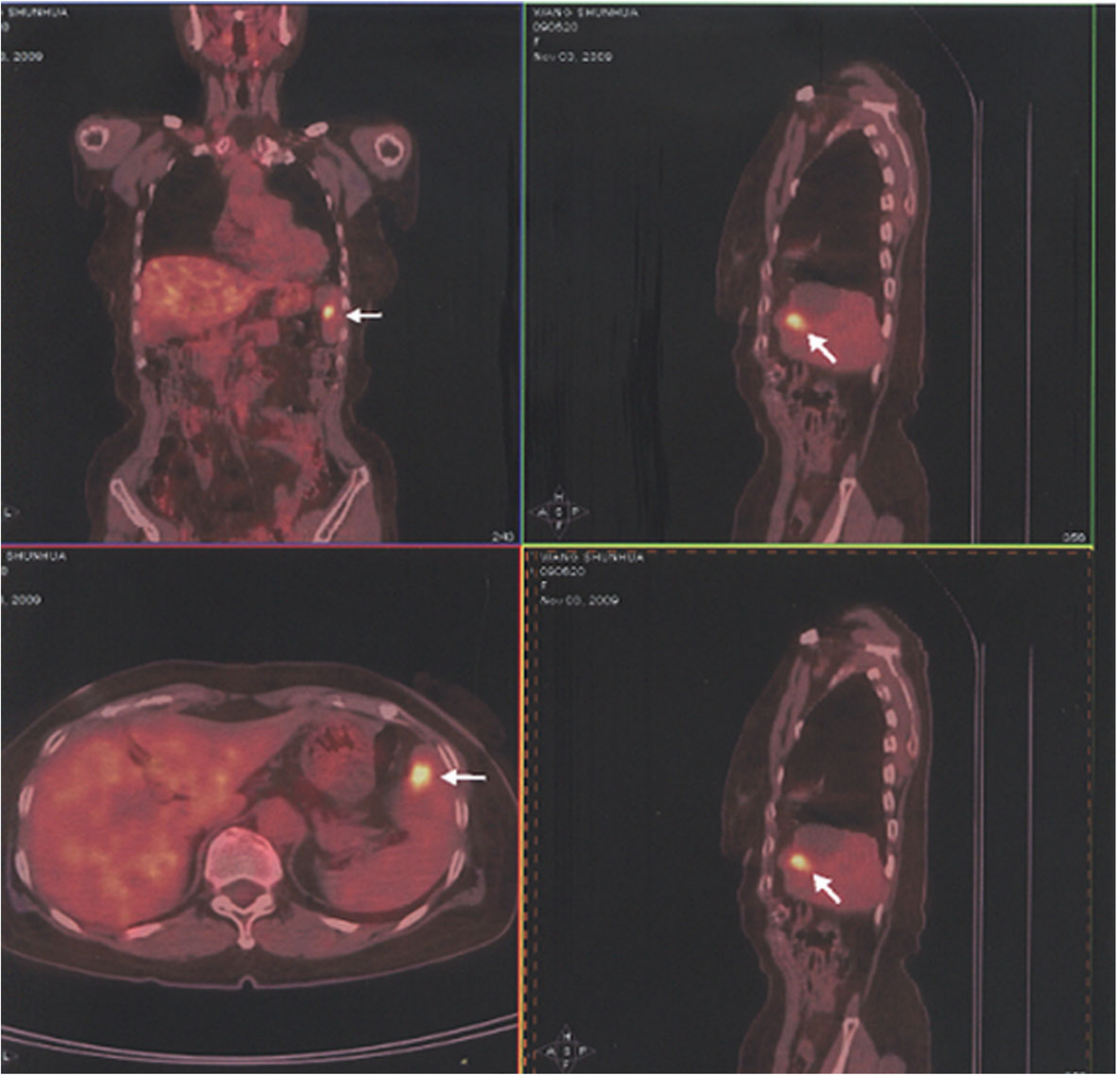
The recurrent lesions was found in the spleen by PET examination when the value of CA125 is 14.5 U/ml

**Fig. 2:**
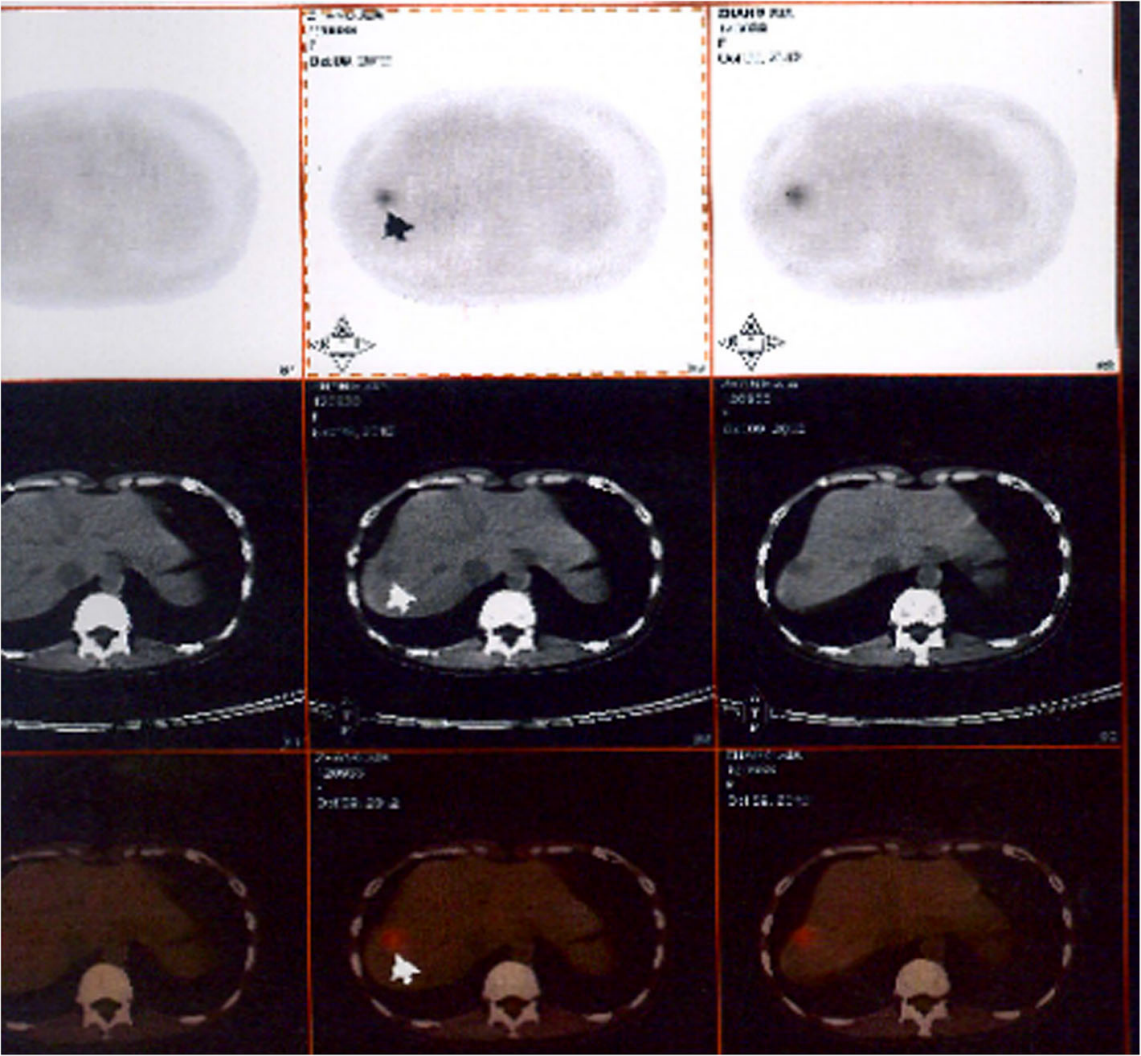
The recurrent lesions was found in the liver by PET examination when the value of CA125 is 13.5 U/ml

**Fig. 3:**
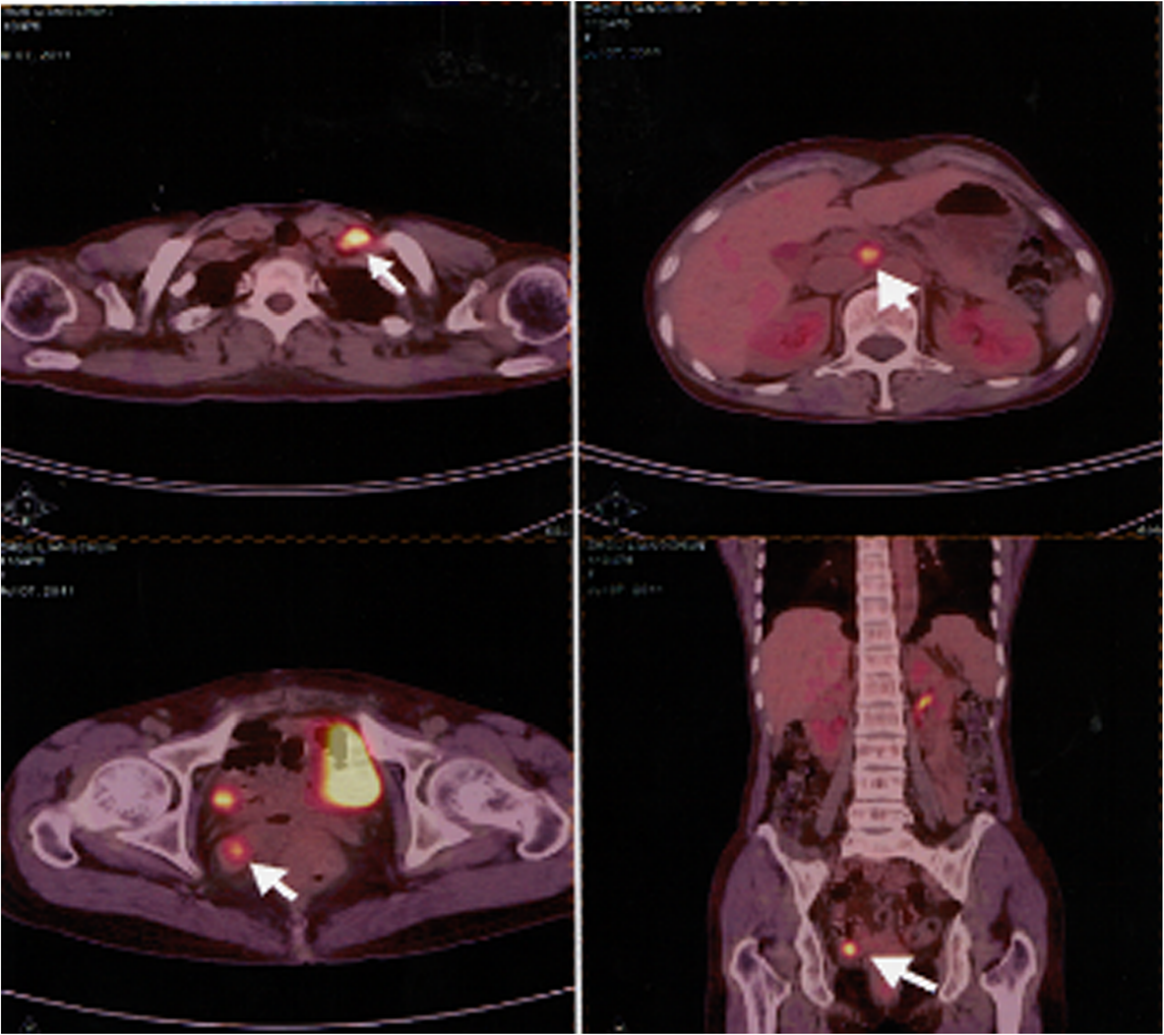
The recurrent lesions was found in the pelvic by PET examination when the value of CA125 is 20.9 U/ml

There were 46 cases of recurrent patients in the total proportion of epithelial ovarian carcinoma patients (13.5%). The follow-up time is 12–144 months, and the median time is 76 months. The dynamics of changes in CA125 levels is 10.2–82.8 U/ml, the median is 29.7 U/ml, and the mean ± standard deviation is 31.3 ± 16.2 U/ml (Table 2). The postoperative CA125 values were divided into four groups:≤10 U/ml, 10–20 U/ml, 20–35 U/ml, and ≥35 U/ml (Table 4), the CA125 ≤ 35 U/ml in the total proportion of recurrent postoperative patients of epithelial ovarian carcinoma when found the recurrent lesions is 31/46 (67.4%).. When the value of CA125 is higher than 10 U/ml and continuously increased, general the time from starting elevation of CA125 to recurrences were 2 to 3 months,need to be vigilant and should be combined with imaging examination (PET-CT). Three cases of recurrent patients was found the recurrent lesions by PET examination when the value of CA125 is 14.5 U/ml, 13.5 U/ml, and 20.9 U/ml, and the recurrent lesions was found in the spleen (Fig. 1), in the liver (Fig. 2), and in the pelvic (Fig. 3), respectively, after the second surgery, up to now, the values of CA125 are all less than 10 U/ml and the patients were all alive so far.

## Discussion

Serum CA125 is mainly found in mesothelial cells and Miller tubular epithelial tissues, including peritoneal, pleural, pericardial, fallopian tube, endometrial, and cervical tissues. It acts as an antigen not only for ovarian carcinoma but also for inflammatory response disease that comes from mesothelial cells and Miller tubular epithelial tissues [4]. Postmenopausal women and non-recurrent postoperative patients of gynecological malignant tumor almost do not contract CA125_-_related diseases, such as pelvic inflammatory disease, endometriosis, peritonitis, etc. Therefore, without any complication cases, the range of CA125 is consistent with that of non-recurrent postoperative patients of epithelial ovarian carcinoma.

James reported 11 epithelial ovarian cancer patients whose histological or radiological examination revealed recurrence, their serum CA125 continuously increased three times within the normal range from one to 3 months [5]. In the present study, the median values of CA125 for non-recurrent patients of epithelial ovarian carcinoma, menopausal women and postoperative patients of gynecological malignant tumor are all less than 10 U/ml. The CA125 ≤ 10 U/ml in the total proportion of postmenopausal women is 3134/3175 (98.7%), The CA125 ≤ 10 U/ml in epithelial ovarian carcinoma non-recurrent patients is 275/296 (92.9%), statistical analysis of the CA125 values of non-recurrent postoperative patients of epithelial ovarian carcinoma and postmenopausal women and non-recurrent patients of gynecological malignant tumor showed no significant difference (*P* >0.05). The CA125 in 31 cases of recurrent patients of epithelial ovarian carcinoma whose CA125 ≤ 35 U/ml continuously increased within the normal range before recurrence, consistent with the report. Among recurrent patients, before recurrent the value of CA125 were all less than 10 U/ml and when the CA125 were continuously increased and combined with imaging examination (PET-CT), three cases of recurrent patients was found the recurrent lesions by PET examination when the value of CA125 is 14.5 U/ml, 13.5 U/ml, and 20.9 U/ml, respectively, we found the recurrent lesions early and the patients had the opportunity for surgery, after the second surgery, up to now, the values of CA125 are all less than 10 U/ml and the patients were all alive so far.

Low reported that in 76 cases of postoperative patients with epithelial ovarian carcinoma followed up in 1 year, 68 revealed recurrence by MRI, 24 (35.3%) of which have a CA125 ≤ 35 U/ml [6]. Garcia-Velloso reported that 55 cases of postoperative patients of epithelial ovarian carcinoma revealed recurrence by PET, 11 of which have CA125 ≤ 35 U/ml (11/55, 20.0%) [7]. Crawford reported 106 cases of postoperative patients of epithelial ovarian carcinoma. The median survival time of patients whose CA125 ≤ 10 U/ml is 2968 days, those whose CA125 is between 11 and 20 U/ml is 537 days, and those whose CA125 is between 21 and 30 U/ml is 537 days. They correspondingly proposed that when patients have CA125 ≤ 10U/ml, the recurrence rate is low and prognosis is good [8]. Bese reported 45 cases of postoperative patients of epithelial ovarian carcinoma, of which 28 who had CA125 ≥ 20 U/ml based on gynecological examination and imaging examination did not show recurrence, but the second exploratory surgery found recurrence [9]. Sugiyama reported 62 cases of postoperative patients of epithelial ovarian carcinoma using 20 U/ml as the cut-off value to monitor recurrence and for early detection of recurrent lesions [10]. In comparison, this study examined 46 cases of recurrent postoperative patients of epithelial ovarian carcinoma, 31 of which have CA125 ≤ 35 U/ml (31/46, 67.4%). According to our study, the detection value of CA125 in patients with ovarian epithelial cancer after surgery is necessary, it can be found the recurrent lesions timely, timely treatment, so as not to delay the treatment time. This is different from some scholars’ point of view, some scholars believe that serum CA125 has no clinical value for the follow-up monitoring the recurrence for postoperative patients with epithelial ovarian cancer [3], they established the benefits of early treatment on the basis of increased CA125 concentrations compared with delayed treatment on the basis of clinical recurrence, the result showed no evidence of a survival benefit with early treatment of relapse on the basis of a raised CA125 concentration alone. In their treatment, the patients were only treated by chemotherapy, but not surgery. It is known that ovarian cancer is mainly treated by surgery, our clinical follow-up found that when the serum CA125 value is <35 U/ml, some postoperative patients of epithelial ovarian carcinoma who underwent cytoreductive or radical surgery and chemotherapy show recurrent lesions in gynecological and imaging examinations or in laparotomy exploration and biopsy, early detected the lesions, the patients had the opportunity for surgery, the prognosis were improvement and the survival time were prolonged.

Some scholars believe that women at familial/genetic ovarian cancer risk often undergo screening despite unproven efficacy. each woman has her own CA125 baseline, serum CA125 q3 months, evaluated using a risk of ovarian cancer algorithm (ROCA), detected significant increases above each subject’s baseline, which triggered transvaginal ultrasound, ROCA q3 months had better early-stage sensitivity at high specificity compared with CA125 > 35 U/mL q6/q12 months [11], in our study, the recurrence rate of epithelial ovarian carcinoma in postoperative patients is high, and most cases of recurrence involve large lesions, the time of treatment is also delayed. Patients are not sensitive to chemotherapy drugs, the treatment is ineffective, and the prognosis is poor. If the value of the CA125 for postoperative patients of epithelial ovarian carcinoma between 10 and 35 U/ml indicates a relative risk of recurrence. When the value of CA125 is higher than 10 U/ml and continuously increased, need to be vigilant and should be combined with imaging examination (PET-CT).early detected the lesions, the patients had the opportunity for surgery.

## Conclusions

When the value of CA125 is higher than 10 U/ml and continuously increased, need to be vigilant and should be combined with imaging examination (PET-CT), early detection of recurrent lesions and early retreatment, especially maybe have the opportunity for surgery, this result may improve the prognosis for recurrent patients.

## References

[1] Bast RC, Klug TL, John E (1983). A radioimmunoassay using a monoclonal antibody to monitor the course of epithelial ovarian cancer. N Engl J Med.

[2] Gynecologic Oncology Branch of the Chinese Medical Association (2003). The standard diagnosis and treatment of recurrent ovarian cancer (recommended). Chin J Obstet Gynecol.

[3] Rustin GJ, van der Burg ME, Griffin CL (2010). Early versus delayed treatment of relapsed ovarian cancer (MRC OV05/EORTC 55955): a randomised trial. Lancet.

[4] Zeng J, Yin J, Song X (2016). Reduction of CA125 Levels During Neoadjuvant Chemotherapy Can Predict Cytoreduction to No Visible Residual Disease in Patients with Advanced Epithelial Ovarian Cancer, Primary Carcinoma of Fallopian tube and Peritoneal Carcinoma. J Cancer.

[5] James L, Edward P, John M (2013). Clinical implications of a rising serum CA-125 within the normal range in patients with epithelial ovarian cancer: a preliminary investigation. Gynecol Oncol.

[6] Low RN, Duggan B, Barone RM (2005). Treated ovarian cancer: MR imaging, laparotomy reassessment, and serum CA-125 values compared with clinical outcome at 1 year. Radiology.

[7] Garcia-Velloso MJ, Jurado M, Ceamanos C (2007). Diagnostic accuracy of FDG PET in the follow-up of platinum-sensitive epithelial ovarian carcinoma. Eur J Nucl Med Mol I.

[8] Crawford SM, Peace J (2005). Does the nadir CA125 concentration predict a long-term outcome after chemotherapy for carcinoma of the ovary?. Ann Oncol.

[9] Bese T, Demirkiran F, Arvas M (1997). What should be the cut-off level of serum CA125 to evaluate the disease status before second-look laparotomy in epithelial ovarian carcinoma?. Int J Gynecol Cancer.

[10] Sugiyama T, Nishida T, Komai K (1996). Comparison of CA 125 assays with abdominopelvic computed tomography and transvaginal ultrasound in monitoring of ovarian cancer. BJOG-Int J Obstet GY.

[11] Skates SJ, Greene MH, Buys SS, et al. Early Detection of Ovarian Cancer using the Risk of Ovarian Cancer Algorithm with Frequent CA125 Testing in Women at Increased Familial Risk - Combined Results from Two Screening Trials. Clin Cancer Res. 2017. doi: 10.1158/1078-0432.CCR-15-2750. 10.1158/1078-0432.CCR-15-2750 PMC5726402 28143870

